# Children oral health and parents education status: a cross sectional study

**DOI:** 10.1186/s12903-023-03424-x

**Published:** 2023-10-24

**Authors:** Giuseppe Minervini, Rocco Franco, Maria Maddalena Marrapodi, Marco Di Blasio, Vincenzo Ronsivalle, Marco Cicciù

**Affiliations:** 1 Saveetha Dental College & Hospitals Saveetha Institute of Medical & Technical Sciences, Saveetha University, Chennai, India; 2 Multidisciplinary Department of Medical-Surgical and Dental Specialties, University of Campania Luigi Vanvitelli, Napoli, Italy; 3 Department of Life, Health and Environmental Sciences, University of L’Aquila, 67100 L’Aquila, Italy; 4 Department of Woman, Child and General and Specialist Surgery, University of Campania “Luigi Vanvitelli”, 80121 Naples, Italy; 5 Department of Medicine and Surgery, University Center of Dentistry, University of Parma, 43126 Parma, Italy; 6 Department of Biomedical and Surgical and Biomedical Sciences, Catania University, 95123 Catania, Italy

**Keywords:** Early Childhood Oral Health Impact Scale (ECOHIS), Oral health, Children, Educational level, Employee status, Dentistry

## Abstract

**Introduction:**

Oral diseases are common and affect millions of people worldwide. They can range from mild and easily treatable conditions to more severe and serious diseases. Proper oral hygiene and regular dental monitoring are essential for maintaining good oral health. When it comes to children’s health and well-being, parents’ education level plays a critical role. Research has shown that parents’ higher educational attainment is associated with better health outcomes for their children.

Our aim is to evaluate whether parents’ education level and employment influence children oral health and its impact on the family.

**Methods:**

We enrolled consecutively healthy subjects aged between 0–16 and their parents at the Dental Clinic of the University of Campania “L. Vanvitelli”. The Italian version of the ECOHIS (I-ECOHIS) was administered to parents of the enrolled subjects referred to the Dental Clinic of the University of Campania “L. Vanvitelli”. Linear regression models, adjusted for age and sex, were used to explore the association between parents’ employment or education level and the ECOHIS scores. Statistical significance was accepted when *p* value < 0.05.

**Results:**

We found a significative association of a higher I-ECOHIS total score (coeff. 4.04244; CI 95%: 1.530855–6.554026; *p* = 0,002) and higher I-ECOHIS children section score (coeff. 3.2794; CI 95%: 1.29002–5.268; *P* = 0,002) and the father unemployed status.

We also found that a higher education level of the father was associated with a lower ECOHIS total score (coeff. -1.388; IC 95%: -2.562115—-0.214 *p* = 0.021) and a higher education level of the mother was associated with a lower ECOHIS in children section (coeff. -0.972; IC95%: -1.909356—0.034; *p* = 0.042).

**Conclusions:**

Father unemployed status and a lower educational level for both parents may negatively affect oral health status.

## Introduction

Oral diseases are common and affect millions of people worldwide. They can range from mild and easily treatable conditions to more severe and serious diseases. Proper oral hygiene and regular dental monitoring are essential for maintaining good oral health [1–4].

The most common oral diseases are cavities, gingivitis, periodontal disease and temporomandibular disorders [5]. Proper oral hygiene is essential in preventing and treating these conditions. Brushing and flossing regularly help remove plaque and bacteria from the teeth and gums. Early detection and treatment of oral diseases can help prevent more serious complications.

The Early Childhood Oral Health Impact Scale (ECOHIS) is a questionnaire to elucidate the correlation between parental perceptions of the quality of life of their preschool children and their oral health status [2, 5–8].

Early Childhood Oral Health Impact Scale (ECOHIS) is a tool designed to measure the impact of oral disease on the quality of life of children. The scale was developed by the World Health Organization and the International Association for Dental Research. The main advantage of the ECOHIS is that it provides a comprehensive assessment of a child’s oral health. It allows for the identification of oral health issues early on before they become more serious and potentially more difficult to treat. Therefore, it can be considerable as a very sensitive tool [2, 8].

Decayed, missing and filled dental treatments are an important part of overall oral health. They are essential for preventing the onset of more serious dental issues such as gum disease, tooth decay, and tooth loss. Tooth loss is the most important cause of prosthesis [9–16]. Decayed teeth occur when bacteria and plaque buildup on the teeth, leading to a weakened enamel. This can be treated with a filling, which is a material that is inserted into the cavity to replace the damaged enamel. Filling materials come in a variety of forms, including composite, silver amalgam, and gold [17–20].

When it comes to children’s health and well-being, parents’ education level plays a critical role. Research has shown that parents’ higher educational attainment is associated with better health outcomes for their children. This is likely since parents with higher educational attainment are more likely to have the knowledge, resources, and access to health care that are necessary for promoting optimal health for their children [21]. Parents with higher levels of education are more likely to have the knowledge and skills necessary to effectively communicate with their children about the risks associated with certain behaviors. They are also more likely to be able to provide their children with the resources and support they need to stay safe and healthy. Finally, parents with higher levels of education are more likely to have the financial resources necessary to ensure that their children have access to quality education and health care. This, in turn, can lead to better overall health and well-being for children, which can further reduce their likelihood of engaging in risky behaviors.

Oral diseases have a multi-factorial etiology. The socio-behavioral and environmental factors play a key role in oral health. Many epidemiological studies have revealed that differences in socio-economic status influence oral health. Indeed, the prevalence and severity of oral pathology vary within and between countries depending on socio-economic status [21–24].

Further, a recent systematic review found that school-based oral health promotion programs generally had positive effects on children and adolescents. These programs, conducted in preschools, elementary schools, and high schools, led to improved oral health knowledge, behaviors, status, and quality of life. The study emphasizes the effectiveness of these initiatives, particularly when involving children, teachers, and parents.

Our aim is to evaluate whether parents’ education level and employment influence children oral health and its impact on the family.

## Methods

The study was conducted in accordance with the Declaration of Helsinki, and the protocol was approved by the Ethics Committee of the Institute, University of Campania *Luigi Vanvitelli* [Protocol number: #555/2017; Date: 05/10/2017]. The study protocol was developed, and all subjects gave their written informed consent for inclusion before they participated in the study.

We enrolled consecutively healthy subjects aged between 0–16 and their parents at the Dental Clinic of the University of Campania “L. Vanvitelli”.

The Italian version of the ECOHIS (I-ECOHIS) was administered to parents of the enrolled subjects referred to the Dental Clinic of the University of Campania “L. Vanvitelli”, regardless of age, June and December 2018, in order to evaluate oral health status parents’ perception of their children oral health status. The Italian version was already validated in subjactes aged between 0–16 years as a reliable measure of oral health in a previous study. As the original version, the I-ECOHIS is composed by 13 items divided into two domains: 9 questions explore the impact on children and the 4 the impact on parents.

Inclusion criteria: subjects aged between 0–16 years, availability to fill the questionnaire by children and their parents.

Exclusion criteria: concomitant pathologies of any order and type, to avoid bias due to health status related to other affections than the oral ones.

An operator involved in the study was present during the questionnaire administration in order to help and record the comprehensibility reported by each parent. According to Pahel et al., the I-ECOHIS answers were coded as follows: 0 = never; 1 = hardly ever; 2 = occasionally; 3 = often; 4 = very often; 5 = don ‘t know, and the total scores were calculated as a simple sum of the response codes for the child and family sections separately, ranging from 0 to 36 and 0 to 16, respectively.

We also collected data regarding parents’ employment status and parents’ education level.

### Sample size

An initial statistical evaluation of sample size was performed considering (I) a power of 95%, (II) two-tailed analysis and significance level of 1% (to correct for multiple comparisons), (III) the mean of the total ECHOIS scored by children with employed father (7.98 ± 4.26) and the mean of the total ECHOIS scored by children with unemployed father (10.4) obtained from a population of 43 patients (our preliminary results). This analysis suggested a minimum sample size of 81 individuals per group.

### Statistical analysis

Continuous variables were expressed as mean and standard deviation (SD) or median with range. The prevalence of categorical variables was expressed as a number and percentage.

The outcomes were I-ECOHIS total score, I-ECOHIS children section and I-ECOHIS family section. The outcome variables were considered as continuous variable.

Parents’ employment and educational level were used as predictors. Employment was treated as binary variable (presence or absence of employee at the time of questionnaire administration. Educational level was treated as an ordinal variable: 1, elementary school license, 2, middle school diploma, 3, diploma, 4, degree.

Mann–Whitney test was used to compare mean I-ECOHIS levels between children with employed or unemployed parents. Linear regression models, adjusted for age and sex, were used to explore the association between parents’ employment or education level and the ECOHIS scores.

Statistical analyses were performed using Stata Statistical Software (Release 16, StataCorp LLC, College Station, TX, USA). Statistical significance was accepted when *p* value < 0.05.

## Results

The questionnaire was administered to 184 consecutive Italian-speaking parents/caregivers, irrespective of children age (age range, 2–16 years; mean age, 7,84 ± 3,23 years). 85/184 (46,19%) were male.

The mean I-ECOHIS in the whole population was 9,68 ± 6,53; the mean I-ECOHIS child section was 6,84 ± 5,1; the mean I-ECOHIS family section was 2,91 ± 3,28. The higher I-ECOHIS total score calculated was 24/50, while the higher “child section” and “family section” scores reported were 20/35 and 13/15 respectively. More details in Table 1.

**Table 1:** Results of the questionnaire

		**Children (184)**	**Father (180)**	**Mother (181)**
Age (mean, ds)		7.84 ± 3.23	-	-
Female sex (n, %)		85 (46.19%)	-	-
Unemployed (n, %)		-	17.8%	68.8%
Educational level. years (n, %)	Elementary school certificate	-	5.03%	6.67%
	Middle school certificate		50.84%	46.67%
	High school diploma		29.61%	33.89%
	College degree		14.53%	12.78%
I-ECOHIS (mean, SD)		9.68 ± 6.53	-	-
I-ECOHIS child section (mean, SD)		6.84 ± 5.1	-	-
I-ECOHIS family section (mean, SD)		2.91 ± 3.28	-	-

When we compared the I-ECOHIS and I-ECOHIS sections between children with employed father with those with unemployed father, we found that children with employed father present a lower total ECOHIS score (8.92 ± 5.96vs12.96 ± 7.50; *p* = 0,0087), lower I-ECOHIS children section score (6.237 ± 4.53 vs 9.51 ± 6.65; *p* = 0.019) and a slightly lower, not significative, score in I-ECOHIS family Sect. (2.75 ± 3.23 vs 3.44 ± 3.15; *p* = 0.17).

Conversely, we did not find any difference in the I-ECOHIS score and I-ECOHIS section scores between children with employed mother with those with unemployed mother.

At the regression models, we found a significative association of a higher I-ECOHIS total score (coeff. 4.04244; CI 95%: 1.530855–6.554026; *p* = 0,002) and higher I-ECOHIS children section score (coeff. 3.2794; CI 95%: 1.29002–5.268; *P* = 0,002) and the father unemployed status.

**Fig. 1:**
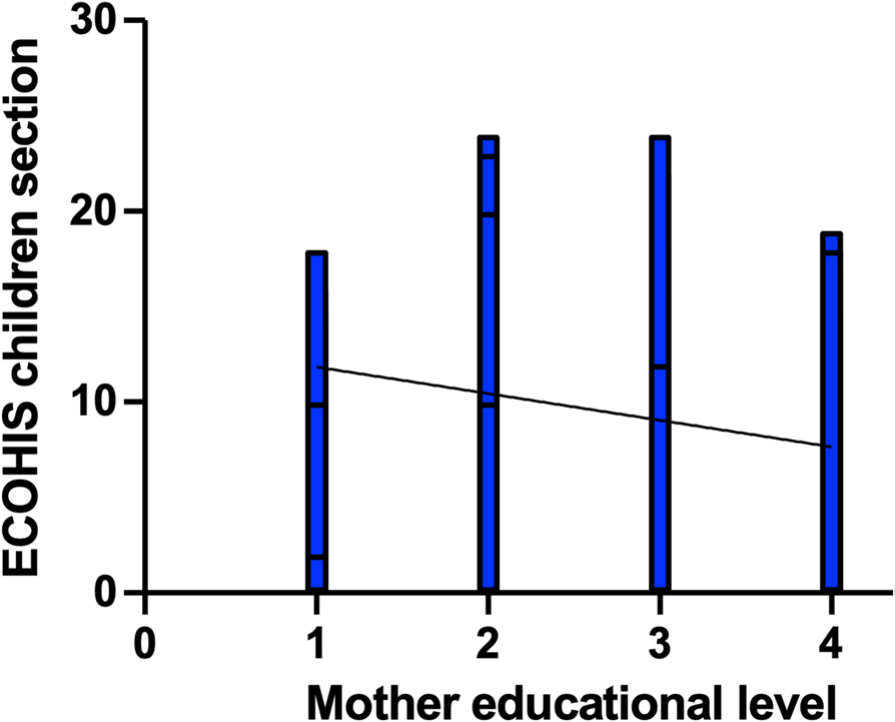
Graph on the level of mother's education

**Fig. 2:**
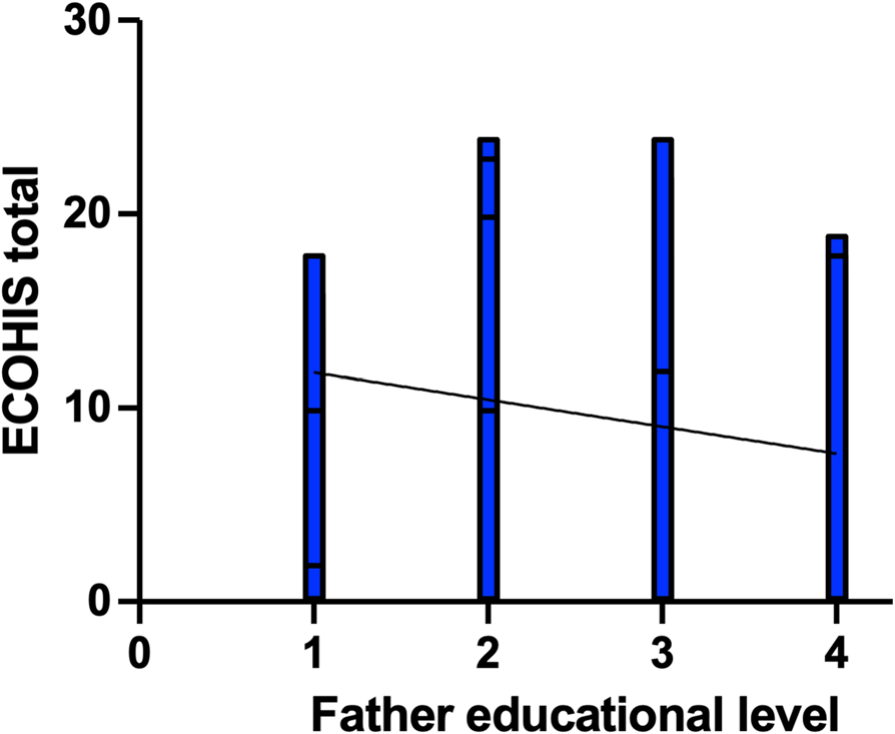
Graph on the level of father's education

We also found that a higher education level of the father was associated with a lower I-ECOHIS total score (coeff. -1.388; IC 95%: -2.562115—-0.214 *p* = 0.021) and a higher education level of the mother was associated with a lower I-ECOHIS in children section (coeff. -0.972; IC95%: -1.909356—0.034; *p* = 0.042). The regression models exploring the association between educational level and ECOHIS score are depicted in Figs. 1 and 2.

## Discussion

In this study we have evaluated whether parents’ educational level and employment status influence the oral health condition of their children.

We found that an unemployed status for the father and a lower educational level for both parents may negatively affect oral health status [18, 19, 25].

There is a clear relationship between parents' educational level and their ability to prevent their children from being involved in risky behaviors. Studies have shown that children of parents with higher levels of education are more likely to avoid risky behaviors than those whose parents have lower levels of education. There are several reasons why this relationship exists. First, parents with higher levels of education are more likely to have the knowledge and skills necessary to effectively communicate with their children about the risks associated with certain behaviors. They are also more likely to be able to provide their children with the resources and support they need to stay safe and healthy. Finally, parents with higher levels of education are more likely to have the financial resources necessary to ensure that their children have access to quality education and health care. This, in turn, can lead to better overall health and well-being for children, which can further reduce their likelihood of engaging in risky behaviors.

Research has shown that children of parents with higher levels of education have lower rates of obesity and childhood asthma. This is likely due to the fact that parents with higher levels of education are more likely to have the knowledge and resources to ensure their children are eating nutritiously and exercising regularly [26–30]. They are also more likely to have access to health care resources that can help diagnose and treat asthma, as well as access to preventative preventive health care, such as preventive treatment and regular check-ups. In addition, research has shown that children of parents with higher levels of education are less likely to be exposed to environmental hazards, such as air pollution or lead-based paint [18, 31, 32]. Parents with higher levels of education are more likely to be aware of environmental hazards and take steps to reduce their children’s exposure to them. Finally, research has shown that children of parents with higher levels of education are more likely to receive appropriate mental health care and treatment. Parents with higher levels of education are more likely to recognize the signs and symptoms of mental health issues [5] and access appropriate care and treatment for their children [26–28]. Overall, parents’ higher educational attainment is associated with better health outcomes for their children. Parents with higher levels of education are more likely to have the knowledge, resources, and access to health care that are necessary for promoting optimal health for their children. Moreover, they are more likely to be aware of environmental hazards and take steps to reduce their children’s exposure to them, as well as recognize the signs and symptoms of mental health issues [5] and access appropriate care and treatment for their children [5, 19, 20, 33].

Oral diseases have a multi-factorial etiology. The socio-behavioral and environmental factors play a key role in oral health. Many epidemiological studies have revealed that differences in socio-economic status influence oral health. Indeed, the prevalence and severity of oral pathology vary within and between countries depending on socio-economic status [21–24].

In numerous Asian and African countries poor oral health is unequally distributed, with a higher prevalence of oral pathology in socially disadvantaged communities [34].

Edelstein reviewed numerous national surveys exploring oral health of children between 2–4 years and 6–8 years. He found that children of parents with less than high school education had the highest caries experience compared to children of parents with higher education levels [35–37].

Another study found that children that children who had parents with < 8 years of education experienced higher levels of caries than the children of more educated parents [38].

These findings are in line with the results of our study. Herein, we found that a lower educational level of both parents is associated with a higher likelihood of presenting oral health issues. The association between the unemployed status of the father and the higher scores at ECOHIS could be indirectly linked to the relationship that occurs between a lower educational level and an unemployed status.

Understanding oral health and educational backgrounds can be complex due to various factors. Diagnosing oral health issues often relies on subjective patient descriptions, which may not always accurately convey the severity of symptoms. Limited access to dental care, especially among disadvantaged groups, can result in delayed diagnoses, allowing conditions to worsen. Diagnosing oral problems in children can be challenging due to their limited ability to communicate symptoms effectively. Furthermore, oral diseases often manifest subtly initially, making them difficult to detect without comprehensive examinations. Dental anxiety and fear can deter patients from undergoing necessary assessments, further complicating diagnosis. Assessing a patient's educational level can be equally challenging. Many patients lack formal education documentation, and language barriers can hinder accurate assessments, especially in multilingual settings. Patients may not always provide precise information about their educational history, and limited literacy skills can affect their ability to convey their background accurately. Non-standard educational forms, cultural norms, and privacy concerns add further complexity to evaluating educational levels. To navigate these challenges, healthcare providers must adopt patient-centered and culturally sensitive approaches, ensuring effective communication and trust-building to facilitate accurate diagnoses and educational assessments.

The persistence of oral health inequalities demands preventive efforts toward communities at higher risk, through policies that are both proportionate (targeting the least advantaged) and universal (accessible to all). In this direction, the increasing cost and inequitable access to quality healthcare, coupled with the merger of the information technology [39] and health service sectors, has given rise to the modern field of telemedicine [40, 41]. The use of telecommunications technology to exchange dental information between distant locations in order to provide dental care or consultation is termed ‘teledentistry’. This mode of delivery of dental care has been found to be feasible, efficient and cost effective. It provides an opportunity to reach out to patients in remote and underserved areas. Telemedicine is the use of telecommunications technology to exchange medical information between distant locations in order to provide healthcare services. Teledentistry is a subspecialty of telemedicine that involves the use of telecommunications technology to exchange dental information between distant locations in order to provide dental care or consultation. The use of teledentistry has been found to be feasible, efficient and cost effective. It provides an opportunity to reach out to patients in remote and underserved areas. A study conducted by the American Dental Association found that patients who used teledentistry services were highly satisfied with the care they received. The study also found that teledentistry is a cost-effective way to provide dental care [42]. Telemedicine is therefore receiving more attention as a potential healthcare delivery alternative, particularly for chronic illnesses.

### Limitations

It's important to acknowledge some limitations of the present report. Firstly, this study is observational in nature, which means it can establish associations but not causation. Secondly, the study was conducted in a specific geographical area and may not fully represent broader populations. Additionally, the study relied on self-reported data from parents, which could introduce response bias. Further research with larger and more diverse samples, as well as longitudinal designs, is needed to confirm these findings and explore potential causal relationships. Moreover, the study did not consider other potential confounding factors that could influence oral health, such as dietary habits and access to dental care. Finally, the impact of teledentistry on addressing oral health inequalities, mentioned in the discussion, warrants further investigation to assess its feasibility and effectiveness in different contexts.

## Conclusions

In conclusion, this study found that parental education level and employment status significantly influence their children's oral health. Children with employed fathers had lower oral health impact scores, while lower parental education levels correlated with higher oral health burdens. These findings reinforce the role of education and employment in guiding children away from risky behaviors and ensuring access to healthcare.

Oral diseases have complex causes, often tied to socio-economic factors. Disparities in oral health persist among different communities, emphasizing the need for targeted healthcare policies. Telemedicine, including teledentistry, holds promise in bridging healthcare gaps.

In summary, this research underscores the importance of parental socio-economic factors in children's oral health outcomes. Comprehensive healthcare policies, along with innovative approaches like telemedicine, can help reduce disparities and improve the well-being of children from diverse backgrounds.

## Data Availability

The corresponding author will have access to the data that were the basis for this article.

## Abbreviations

ECOHIS: Early Childhood Oral Health Impact Scale
WHO: World Health Organization
I-ECOHIS: Italian version of the Early Childhood Oral Health Impact Scale
SD: Standard Deviation
CI: Confidence Interval

## References

[1] Shay B, Ben Ami O, Ianculovici DL, Zini A, Ianculovici C, Almoznino G (2019). Oral health-related quality of life in patients with disorders of nutrition. J Oral Rehabil.

[2] Bekes K, Solanke C, Waldhart T, Priller J, Stamm T (2021). Effect of method of administration on the oral health–related quality of life assessment using the Early Childhood Oral Health Impact Scale (ECOHIS-G). Clin Oral Investig.

[3] Rodrigues JA, Azevedo CB, Chami VO, Solano MP, Lenzi TL (2020). Sleep bruxism and oral health-related quality of life in children: A systematic review. Int J Paediatr Dent.

[4] Minervini G, Franco R, Marrapodi MM, Crimi S, Badnjević A, Cervino G (2023). Correlation between Temporomandibular Disorders (TMD) and Posture Evaluated trough the Diagnostic Criteria for Temporomandibular Disorders (DC/TMD): A Systematic Review with Meta-Analysis. J Clin Med.

[5] Li S, Malkinson S, Veronneau J, Allison PJ (2008). Testing responsiveness to change for the early childhood oral health impact scale (ECOHIS). Community Dent Oral Epidemiol.

[6] Novaes TF, Pontes LRA, Freitas JG, Acosta CP, Andrade KCE, Guedes RS (2017). Responsiveness of the Early Childhood Oral Health Impact Scale (ECOHIS) is related to dental treatment complexity. Health Qual Life Outcomes..

[7] Zaror C, Atala-Acevedo C, Espinoza-Espinoza G, Muñoz-Millán P, Muñoz S, Martínez-Zapata MJ (2018). Cross-cultural adaptation and psychometric evaluation of the early childhood oral health impact scale (ECOHIS) in chilean population. Health Qual Life Outcomes..

[8] Lee VHK, Grant CG, Mittermuller BA, et al. Association between early childhood oral health impact scale (ECOHIS) scores and pediatric dental surgery wait times. BMC Oral Health. 2020;20:285. 10.1186/s12903-020-01263-8. https://bmcoralhealth.biomedcentral.com/articles/10.1186/s12903-020-01263-8#citeas. 10.1186/s12903-020-01263-8 PMC7568462 33069219

[9] Shaikh M, Alnazzawi A, Habib S, Lone M, Zafar M (2021). Therapeutic Role of Nystatin Added to Tissue Conditioners for Treating Denture-Induced Stomatitis: A Systematic Review. Prosthesis.

[10] Ahmed N, Humayun M, Abbasi M, Jamayet N, Habib S, Zafar M (2021). Comparison of Canine-Guided Occlusion with Other Occlusal Schemes in Removable Complete Dentures: A Systematic Review. Prosthesis.

[11] D’Addazio G, Xhajanka E, Cerone P, Santilli M, Rexhepi I, Caputi S (2021). Traditional Removable Partial Dentures versus Implant-Supported Removable Partial Dentures: A Retrospective Observational Oral Health-Related Quality-of-Life Study. Prosthesis.

[12] Pugliese A, Cataneo E, Fortunato L (2021). Construction of a Removable Partial Denture (RPD): Comparison between the Analog Procedure and the Selective Laser Melting Procedure. Prosthesis.

[13] Mangone E, Cataneo E, Fortunato L, Cresti L (2021). Functional Removable Prosthetic Rehabilitation Using the Electronic Condylograph: A Case Report. Prosthesis.

[14] Minervini G, Franco R, Marrapodi MM, Fiorillo L, Cervino G, Cicciù M (2023). Economic inequalities and temporomandibular disorders: A systematic review with meta-analysis. J Oral Rehabil.

[15] Minervini G, Franco R, Marrapodi MM, Ronsivalle V, Shapira I, Cicciù M. Prevalence of temporomandibular disorders in subjects affected by Parkinson disease: A systematic review and metanalysis. J Oral Rehabil. 2023;50(9):877–85. 10.1111/joor.13496. https://pubmed.ncbi.nlm.nih.gov/37183340/. https://onlinelibrary.wiley.com/doi/10.1111/joor.13496. 10.1111/joor.13496 37183340

[16] Di Cosola M, Cazzolla AP, Charitos IA, Ballini A, Inchingolo F, Santacroce L (2021). Candida albicans and Oral Carcinogenesis. A Brief Review Journal of Fungi.

[17] Behbahanirad A, Joulaei H, Jamali J, Golkari A, Bakhtiar M (2021). Dimensional structure of the early childhood oral health impact scale. Iran J Med Sci.

[18] Chaffee BW, Rodrigues PH, Kramer PF, Vítolo MR, Feldens CA (2017). Oral health-related quality-of-life scores differ by socioeconomic status and caries experience. Community Dent Oral Epidemiol.

[19] Naidu RS, Nunn JH, Pahel B, Niederman R (2021). Editorial: Promoting Oral Health in Early Childhood: The Role of the Family, Community and Health System in Developing Strategies for Prevention and Management of ECC. Front Public Health.

[20] Muhoozi GKM, Atukunda P, Skaare AB, Willumsen T, Diep LM, Westerberg AC (2018). Effects of nutrition and hygiene education on oral health and growth among toddlers in rural Uganda: follow-up of a cluster-randomised controlled trial. Tropical Med Int Health.

[21] Levin KA, Davies CA, Topping GVA, Assaf AV, Pitts NB (2009). Inequalities in dental caries of 5-year-old children in Scotland, 1993–2003. Eur J Public Health.

[22] Pattussi MP, Marcenes W, Croucher R, Sheiham A (2001). Social deprivation, income inequality, social cohesion and dental caries in Brazilian school children. Soc Sci Med.

[23] Shiboski CH, Gansky SA, Ramos-Gomez F, Ngo L, Isman R, Pollick HF (2003). The association of early childhood caries and race/ethnicity among California preschool children. J Public Health Dent.

[24] Christensen LB, Twetman S, Sundby A (2010). Oral health in children and adolescents with different socio-cultural and socio-economic backgrounds. Acta Odontol Scand.

[25] Guney SE, Araz C, Tirali RE, Cehreli SB (2018). Dental anxiety and oral health-related quality of life in children following dental rehabilitation under general anesthesia or intravenous sedation: A prospective cross-sectional study. Niger J Clin Pract.

[26] Silveira Schuch H, Venâncio Fernandes Dantas R, Menezes Seerig L, S Santos I, Matijasevich A, J D Barros A, Glazer Peres K, Peres MA, Demarco FF. Socioeconomic inequalities explain the association between source of drinking water and dental caries in primary dentition. J Dent. 2021;106:103584. 10.1016/j.jdent.2021.103584. https://pubmed.ncbi.nlm.nih.gov/33465449/. https://www.sciencedirect.com/science/article/abs/pii/S0300571221000051?via%3Dihub. 10.1016/j.jdent.2021.103584 33465449

[27] Armfield JM, Mejía GC, Jamieson LM. Socioeconomic and psychosocial correlates of oral health. Int Dent J. 2013;63(4):202–9. Available from: http://www.ncbi.nlm.nih.gov/pubmed/23879256. Cited 2022 Dec 11. 10.1111/idj.12032 PMC9375003 23879256

[28] Najafi F, Rezaei S, Hajizadeh M, Soofi M, Salimi Y, Kazemi Karyani A, et al. Decomposing socioeconomic inequality in dental caries in Iran: cross-sectional results from the PERSIAN cohort study. Arch Public Health. 2020 Aug 18;78(1):75. Available from: http://www.ncbi.nlm.nih.gov/pubmed/36155106. Cited 2022 Dec 11. 10.1186/s13690-020-00457-4 PMC7436972 32832079

[29] Shen A, Bernabé E, Sabbah W (2020). The socioeconomic inequality in increment of caries and growth among Chinese children. Int J Environ Res Public Health.

[30] Cantore S, Mirgaldi R, Ballini A, Coscia MF, Scacco S, Papa F (2014). Cytokine Gene Polymorphisms Associate with Microbiogical Agents in Periodontal Disease: Our Experience. Int J Med Sci.

[31] Mafla AC, Moran LS, Bernabe E (2020). Maternal oral health and early childhood caries amongst low-income families. Community Dent Health.

[32] Venezia P, Ronsivalle V, Rustico L, Barbato E, Leonardi R, Lo GA (2022). Accuracy of orthodontic models prototyped for clear aligners therapy: A 3D imaging analysis comparing different market segments 3D printing protocols. J Dent.

[33] Fong Lai S Hiu, Wun Wong M Kok, Wong HM, Yung Yiu C Kar (2017). Parental oral health literacy of children with severe early childhood caries in Hong Kong. Eur J Paediatr Dent.

[34] Waxman A (2003). Prevention of chronic diseases: WHO global strategy on diet, physical activity and health. Food Nutr Bull.

[35] Edelstein BL (2002). Disparities in oral health and access to care: findings of national surveys. Ambul Pediatr.

[36] Minervini G, Franco R, Marrapodi MM, Fiorillo L, Cervino G, Cicciù M. Prevalence of temporomandibular disorders (TMD) in pregnancy: A systematic review with meta-analysis. J Oral Rehabil. 2023;50(7):627–634. 10.1111/joor.13458. https://pubmed.ncbi.nlm.nih.gov/37021601/. https://onlinelibrary.wiley.com/doi/10.1111/joor.13458. 10.1111/joor.13458 37021601

[37] Di Stasio D, Romano A, Gentile C, Maio C, Lucchese A, Serpico R, Paparella R, Minervini G, Candotto V, Laino L. Systemic and topical photodynamic therapy (PDT) on oral mucosa lesions: an overview. J Biol Regul Homeost Agents. 2018;32(2 Suppl. 1):123–6. 29460529

[38] Oliveira LB, Sheiham A, Bönecker M (2008). Exploring the association of dental caries with social factors and nutritional status in Brazilian preschool children. Eur J Oral Sci.

[39] Cicciù M, Fiorillo L, D’Amico C, Gambino D, Amantia EM, Laino L, et al. 3D digital impression systems compared with traditional techniques in dentistry: A recent data systematic review. Materials. 2020;13(8). 10.3390/ma13081982 PMC7215909 32340384

[40] Ricciardi D, Casagrande S, Iodice F, Orlando B, Trojsi F, Cirillo G (2021). Myasthenia gravis and telemedicine: a lesson from COVID-19 pandemic. Neurol Sci.

[41] Spina E, Tedeschi G, Russo A, Trojsi F, Iodice R, Tozza S (2022). Telemedicine application to headache: a critical review. Neurol Sci.

[42] Spina E, Trojsi F, Tozza S, Iovino A, Iodice R, Passaniti C (2021). How to manage with telemedicine people with neuromuscular diseases?. Neurol Sci.

